# The Role of Forgetting in Undermining Good Intentions

**DOI:** 10.1371/journal.pone.0079091

**Published:** 2013-11-13

**Authors:** Kristina R. Olson, Andrea S. Heberlein, Elizabeth Kensinger, Christopher Burrows, Carol S. Dweck, Elizabeth S. Spelke, Mahzarin R. Banaji

**Affiliations:** 1 Department of Psychology, Yale University, New Haven, Connecticut, United States of America; 2 Department of Psychology, Boston College, Boston, Massachusetts, United States of America; 3 Department of Psychology, University of Connecticut, Storrs, Connecticut, United States of America; 4 Department of Psychology, Stanford University, Stanford, California, United States of America; 5 Harvard University, Cambridge, Massachusetts, United States of America; University of Tokyo, Japan

## Abstract

Evaluating others is a fundamental feature of human social interaction–we like those who help more than those who hinder. In the present research, we examined social evaluation of those who not only intentionally performed good and bad actions but also those to whom good things have happened (the lucky) and those to whom bad things have happened (the unlucky). In Experiment 1a, subjects demonstrated a sympathetic preference for the unlucky. However, under cognitive load (Experiment 1b), no such preference was expressed. Further, in Experiments 2a and 2b, when a time delay between impression formation (learning) and evaluation (memory test) was introduced, results showed that younger (Experiment 2a) and older adults (Experiment 2b) showed a significant preference for the lucky. Together these experiments show that a consciously motivated sympathetic preference for those who are unlucky dissolves when memory is disrupted. The observed dissociation provides evidence for the presence of conscious good intentions (favoring the unlucky) and the cognitive compromising of such intentions when memory fails.

## Introduction

Humans have a basic need to understand and interact with those around us. As a result, we are not mindless observers; rather we actively form impressions of others, deciding whom we do and do not like. Not surprisingly, one factor we weigh is a person’s previous intentional actions. That is, we are more likely to favor someone who volunteers for the PTA or donates books to the library over someone who cheats on his taxes or cuts in line at the grocery store. Even 3 month old infants show this basic preference–preferring helpful agents to harmful agents [1]. In contrast to intentional acts such as these, events that are outside human control–events we might call lucky or unlucky–seem less appropriate as bases for our evaluations. Whether Jane’s house is destroyed by a tornado or Joe wins the lottery tells us nothing about the inherent worth of either person; the randomness of these events signals that they could have happened to anybody and therefore, rationally, we should not use them as the basis for judgment.

In the current research, we test the hypothesis that two processes contribute to people’s evaluations of lucky and unlucky individuals: a more automatic tendency to favor the lucky over the unlucky, and a top-down effort to overcome this tendency. According to this hypothesis, we are less successful in overcoming the automaticity of the first tendency (and therefore more likely to prefer lucky people over unlucky people) when under cognitive load or when declarative memory for the event details is compromised. To examine this hypothesis, we asked people to evaluate lucky targets, unlucky targets, intentional good actors and intentional bad actors. They made these evaluations immediately with no load (Experiment 1a), under cognitive load (Experiment 1b), and after a delay (Experiments 2a and 2b). By investigating the effects of cognitive load and delay on people’s evaluations, we hoped to limit conscious, deliberative processing such as effortful empathy or self-presentation concerns; we predicted that this would reveal a relative preference for lucky individuals.

### The Luck Preference

The tendency to favor the lucky over the unlucky is predicted by just world beliefs [2], associative mechanisms [3], or both. Just World Theory argues that in order to avoid the psychological threat of a chaotic and unpredictable world, people develop the beliefs that “good things happen to good people” and “bad things happen to bad people.” These beliefs lead to favoring the lucky and disparaging the unlucky [2]. People may also come to favor the lucky because of associative links between lucky or unlucky events and the people experiencing them. That is, they may engage in what might be called “affective tagging” or the spreading of affect from an event to the target of that event [3].

Importantly, Lerner [4] has argued that the tendency to show just world thinking emerges most clearly when people are under high load, cannot escape a situation, or are otherwise pressured. In contrast, in situations of unlimited time and cognitive resources, people’s explicit moral reasoning, empathy, or reliance on egalitarian beliefs are more likely to guide judgments and behavior, he argues. Therefore, despite our prediction that people may generally favor the lucky over the unlucky, we predict that adults also know, at least explicitly, that it is critical to evaluate people based primarily on their intentional actions, and not on the lucky or unlucky things that happen to them. We predict that when engagement of conscious cognition and regulatory processes are possible, people will likely recognize that the effects of luck are dissociable from the effects of effort and intention, and therefore largely irrelevant in evaluating the value of a person. This is likely to lead to either equivalent evaluations of lucky and unlucky targets, or even higher evaluations of the unlucky. Following this reasoning, when people are tested under cognitive constraints, such as under conditions of cognitive load or diminished explicit memory, we predict that conscious processing such as egalitarian beliefs and/or impression management concerns would have less of an opportunity to unfold. This prediction is consistent with observations in young children, who presumably engage in less impression management [5], and who show a preference for those who are lucky over those who are unlucky [3], [6]. Specifically, in adults, we predict that a luck preference would be observed in the performance of an evaluation task while under cognitive constraints.

We tested our two-part hypothesis–that adults’ explicit attitudes toward the lucky and unlucky may be influenced by conscious, deliberative processes, and that if these processes can be removed, adults will prefer the lucky to the unlucky– using two approaches: cognitive load and reduced explicit memory (see [7] for a similar approach in the domain of cognitive dissonance).

#### Cognitive load

A signature feature of human cognition is that it is limited: humans can attend to and process a limited amount of information at a given time. Researchers can exploit this feature to understand many aspects of cognition and social cognition. A common cognitive load task involves requiring the completion of two tasks–one more deliberative and one more automatic–at once. This strategy is known to reduce the ability to engage in deliberative tasks, while allowing more automatic processes to remain relatively unaffected (e.g., [8]–[11]). For example, Gilbert and colleagues [12] hypothesized that people automatically draw inferences about others (e.g., the woman looks sad… she is a depressive person), and only subsequently correct those inferences based on the context (she is sad only because her dog just died, so she may not be a sad person in general). Under cognitive load participants drew the initial inference, but did not correct it in response to contextual cues as they did in the absence of load.

In the case of evaluations of the lucky and unlucky, we tested whether people would show a luck preference in the control condition of no load (Experiment 1a). Then, we tested whether the addition of cognitive load, or the reduced ability to engage in conscious processing, would reduce the tendency to favor the unlucky (Experiment 1b).

#### Memory

Manipulating memory, and specifically, reducing people’s ability to explicitly remember information, may similarly reduce the impact of conscious processing on an underlying automatic evaluation, such as a tendency to favor the lucky. There is considerable evidence supporting a dissociation between evaluations of individuals and the explicit retention of information about those individuals. In one review, Hastie and Park [13] noted that while there are experimental conditions under which memory and evaluations can be linked, it is far more common to observe people using “on-line processing” during which they are forming impressions without consciously remembering the attributes leading to that impression. Importantly, on-line processing has been demonstrated to be associated with higher certainty in one’s social evaluations, as well as a stronger attitude-behavior correspondence, such as selection of a favorite political candidate [14]–[15].

The current work builds on a series of classic studies demonstrating evidence of this dissociation between memory and evaluations. For example, Johnson, Kim, and Risse [16] tested patients with Korsakoff’s amnesia, presenting them with two faces, each paired with a story about an individual–one who had performed a series of good, noble acts (e.g., saving someone’s life) and one who had performed a series of bad, immoral acts (e.g., abusing his wife). At three time points ranging from two hours to twenty days later, the patients were presented with the two faces and asked whom they liked better and who was nicer. Remarkably, despite recalling almost nothing about the individuals, the majority of patients (78%-89% across sessions) liked the “good” guy better than the “bad” guy. These patients had formed affective evaluations of the targets and had retained this affective information associated with the faces, despite explicitly forgetting the information that led to these affective evaluations.

A similar study by Tranel and Damasio [17] found that an amnesic patient with extensive neural damage, including bilateral hippocampi and amygdalae, preferred to interact with an experimenter who had been consistently nice on several previous days of testing, over a neutral experimenter; the same patient also showed a preference for the neutral experimenter over one who had been curt and unfriendly over several previous days of testing. All of these preferences occurred despite the patient’s failure to explicitly recall having met the experimenters before. Finally, in a more recent demonstration, Todorov and I. Olson [18] showed that an amnesic patient with bilateral hippocampal damage retained affective associations with a series of faces that had been paired with single sentences describing aggressive, disgusting, or kind (vs. neutral) actions.

Conceptually similar results have been found with neurologically intact populations. Somerville, Wig, Whalen and Kelley [19] conducted a study in which neurologically-healthy adults were exposed to faces of individuals along with positive phrases (Emily helps the homeless), negative phrases (Bob is a deadbeat dad), neutral phrases (Eric likes carrots) or no phrases. Two weeks after seeing the phrases and faces, participants were scanned while performing a simple old/new memory task for the faces. Somerville et al. [19] found that emotional contexts (both positive and negative) were associated with more right amygdala activation than neutral contexts, and that this effect existed irrespective of whether participants could explicitly recall the context or not. This finding suggests that the targets were associated with a valence that remained even after the context was forgotten.

The studies reviewed so far have focused exclusively on later evaluations of targets who have performed intentional good and bad actions. To our knowledge only one existing study has asked about social evaluations of valenced targets who did not perform intentional actions after a delay. Li, Spitzer, and Olson [20], presented 4 and 5 year old children with targets who, for an unknown reason, received differential amounts of a resource (playdough). The authors found that in an immediate evaluation task, children indicated that they preferred the person who had more resources. The critical comparison was with a condition in which another group of children simply saw the targets in passing but were not asked to make any evaluations until after a delay. Several minutes after viewing the targets, during which they forgot who had more playdough, these children showed a preference for the target who had more resources previously.

All of these studies suggest that people can and do associate affective information with people, based on even minimal information about the targets’ actions or current conditions [21]; that they form these associations even without being instructed to do so; and that they rely on the retained affective information even after forgetting the information that led to the initial affective evaluation. Our hypothesis is that the valence of lucky and unlucky events may be similar to the valence associated with intentional actions in affecting evaluations of the people tied to those events. We predict that this valence informs evaluations of those targets, even without explicit memory for an individual’s lucky or unlucky status.

### The Current Work

In sum, our primary prediction is that people have an underlying tendency to favor the lucky, but that this tendency is reduced and even reversed by controlled processes (e.g., impression management, demand effects, effortful or controlled empathy, egalitarian concerns, etc). We predict that under conditions that allow these conscious processes (unlimited time, unlimited resources), adults will show no preference, or that they may even overcorrect, leading them to favor the unlucky (Experiment 1a). We predict that this tendency to favor the unlucky will be reduced or eliminated when participants are asked to simultaneously complete a second task that requires the use of additional cognitive resources because the conscious processes will be less active (Experiment 1b). Finally, we predict that if we create conditions under which adults can no longer explicitly recall who was lucky and who was unlucky, they will actually favor the lucky (Experiments 2a and 2b). We tested this last prediction in both younger adults (Experiment 2a) and older adults (Experiment 2b) because older adults have been shown to rely more heavily on affect when making decisions (see [22] for a recent review) and to have less effective retention of episodic information (see [23] for a recent review), factors likely to increase the luck preference even more.

## Experiment 1a and 1b

### Evaluations of the Lucky and Unlucky under Limited and Unlimited Cognitive Resources

Experiments 1a and 1b were designed to assess whether adults demonstrate the luck preference in a situation of unlimited vs. limited time and cognitive resources. In previous work, 5–7-year-old children evaluated the lucky more favorably than the unlucky under conditions of unlimited cognitive resources [6]. Considerable research suggests that concerns with social desirability and attempts to control bias emerge after this age [24]–[25] leading to the prediction that adults might actually report the opposite preference–favoring those who are unlucky. When adults’ cognitive resources are limited, social desirability, cognitive control, and even empathy are predicted to be less active, thereby reducing the likelihood that adults will favor the unlucky. To test these hypotheses, adult participants were shown photographs of people paired with statements and asked to form an impression of each person (“target”). The statements described an intentional good action or intentional bad action performed by the target, or a lucky (random good) event or unlucky (random bad) event experienced by the target. Participants evaluated each target after the paired statement was removed either under conditions of unlimited cognitive resources (Experiment 1a) or under conditions of limited cognitive resources (Experiment 1b).

Importantly, in the case of limited cognitive resources we wanted to interfere with effortful reasoning processes without interfering with participants’ abilities to read the stimuli or understand them. Therefore we had participants complete the same task as in Experiment 1a while also completing a simultaneous short-term visual memory task (using figures similar to those in [26]). Whereas we expected that cognitive load would alter participants’ evaluations of the unlucky compared to the lucky, we made no such prediction for evaluations of the intentional good and bad targets. As we discussed at the outset, it is likely that people will always favor intentional good to bad targets, unhindered by concerns of impression management or other deliberative processes; after all, even 6 month old babies prefer intentional good to bad targets [27]. Therefore, we predicted that adults would not prefer the unlucky to the lucky under load, but they would prefer intentional good targets over intentional bad targets.

### Methods

#### Participants

Participants included 23 adult college students (M_age_ = 21 years, SD = 3 years, 16 females, 7 males) in Experiment 1a (unlimited cognitive resources) and 23 adult college students (*M_age_* = 20 years, *SD* = 2 years; 14 female, 9 male) in Experiment 1b (limited cognitive resources). All students were recruited at the same university in the Northeastern United States and participated in exchange for a small payment or credit toward a research requirement.

#### Ethics statement

These studies, including the consent forms used, were approved by the human subjects committees at Harvard University, Yale University, and Boston College. All participants signed a consent form before participating. The authors are happy to share the data files described in this paper with any interested researchers.

#### Stimuli

Stimuli included four types of statements: *intentional good acts* (e.g., “Leonard pulled over to help a motorist with his stalled car.”); *intentional bad acts* (e.g., “Matt left the restaurant before paying the bill.”); *lucky events* (e.g., “Andrew was walking when he found $5 on the ground.”); and *unlucky events* (e.g., “Richard was stuck in the elevator for three hours with ten other people.”), as well as photographs of White male faces, all on standardized backgrounds. Two sets of 40 photographs were used such that half of participants saw one set and the other half saw a completely different set. Within each set of photographs, participants saw one of two randomized assignments of photographs with statements. A single race and gender was selected in order to minimize the number of dimensions that varied throughout the experiment and concerns that participants might believe the experiment was about race or gender and therefore spend differential attention on some photographs over others.

These statements were pilot tested (all *N*s≥10 independent raters) separately for valence, level of control, and level of intention. Good, bad, lucky, and unlucky statements were provided such that lucky experiences and intentional good actions were judged as equally positive on average (participants were asked “How good or bad is the outcome or action?”), and unlucky experiences and intentional bad actions were judged to be equally negative, on average. The items varied in terms of extremity from more trivial or mundane events and actions to more extreme, consequential events and actions, with an equal number of both types in all categories. Finally, the intentional actions had been rated by pilot participants as highly and equivalently controlled and intended by the targets, and the lucky/unlucky events were judged to be equivalently low on intention and control. All items are listed in Appendix S1.

#### Procedure

College students were recruited to participate in a short study on “impression formation and multi-tasking.” They were brought into a small testing room one at a time and seated in front of a computer. In Experiment 1a (No Load Condition) they were told that they would see faces of individuals paired with statements, that they should form an impression of each one, and that after forming the impression they would be asked to evaluate the target (“How do you feel about the man you just read about?”) on a scale from 1 (dislike completely) to 6 (like completely). Participants then began the task. They had 10 seconds to form an impression and unlimited time to provide an evaluation. Total participation took approximately 10–15 minutes for 40 face-sentence pairs.

In Experiment 1b (Cognitive Load Condition) participants were told that they would complete two tasks at one time: a social evaluation task (identical to the one in Experiment 1a) and a memory experiment. Before each impression formation/evaluation trial participants were shown a Phillips figure [26] for 2 seconds. They were told that after each impression formation/evaluation trial they would see another such design and their task was to decide if it was the same as the one before that trial or not (it was new on 50% of trials). Participants had unlimited time to evaluate the target and to answer the memory question. This particular cognitive load task was selected because it requires significant cognitive resources (visual-spatial resources) but did not interfere with participants’ ability to read the impression formation/evaluation items. The study took 15–20 minutes to complete.

### Results

#### Omnibus ANOVA

We first computed a 2 (valence: positive vs. negative) X 2 (intentionality/controllability: intentional/controllable vs. unintentional/uncontrollable) repeated-measures ANOVA for Studies 1a and 1b. In Study 1a we found a significant effect of valence, *F*(1, 22) = 132.93, *p*<.001, such that positively-valenced items were rated more positively than negatively-valenced items. There was also a significant effect of intentionality/controllability, *F*(1,22) = 38.45, *p*<.001, such that the unintentional/uncontrollable items were rated more positively than the intentional/controllable ones. Finally, these main effects were qualified by a significant interaction, *F*(1,22) = 109.57, *p*<.001, such that the difference between positively and negatively-valenced items was bigger for the intentional/controllable items than the unintentional/uncontrollable items. In Study 1b we conducted the same omnibus test and found similar results. Again there was a significant effect of valence, *F*(1, 22) = 269.37, *p*<.001, an effect of intentionality/controllability, *F*(1,22) = 49.74, *p*<.001, and these effects were qualified by a significant interaction, *F*(1,22) = 190.81, *p*<.001.

#### The luck preference

Our primary question, however, concerned whether adults, like children, would prefer the lucky to the unlucky under conditions of unlimited time and resources (Experiment 1a) and whether this same effect would be evident under cognitive load (Experiment 1b). We found that adults did *not* prefer the lucky (M = 3.79) under conditions of unlimited time and resources, and in fact that they preferred the *unlucky* (M = 4.00), as indicated by a paired t-test, *t*(22) = 2.53, *p* = .02, see Table 1. For comparison, we also assessed whether they preferred intentionally good actors (*M* = 4.78) to intentionally bad actors (*M* = 2.15), which, not surprisingly, they did, *t*(22) = 11.50, *p*<.001.

**Table 1:** Mean evaluations (and standard deviations) of Good and Bad actors and Lucky and Unlucky targets in each study. Higher numbers indicate greater liking.

	Good	Bad	Lucky	Unlucky
Exp 1A: ImmediateEvaluation	4.78 (.55)	2.15 (.82)	3.79 (.53)	4.00 (.50)
Exp 1B: CognitiveLoad	4.74 (.40)	2.03 (.51)	3.93 (.40)	3.98 (.43)
Exp 2A: Memory - YoungerAdults	3.52 (.59)	3.34 (.57)	3.57 (.59)	3.42 (.58)
Exp 2B: Memory - OlderAdults	4.05 (.85)	2.47 (.65)	3.95 (.62)	2.74 (.79)

Under cognitive load (Experiment 1b), however, participants showed no preference for the unlucky (*M* = 3.98) over the lucky (*M* = 3.93), *t*(22)<1.0, *p*>.30, see Table 1, but did favor the intentional good actors (*M* = 4.74) over the intentional bad actors (*M* = 2.03), *t*(22) = 15.57, *p*<.001, as they had in Experiment 1a.

### Discussion

A central question of this first study concerned whether adults would prefer the lucky to the unlucky under conditions of unlimited time and cognitive resources. As predicted, we found that they did not. Instead, adults indicated that they preferred the *unlucky* to the lucky, but only when they had unlimited cognitive resources during evaluation. When participants were under cognitive load, they showed no preference for the unlucky, despite continuing to show a preference for good over bad targets.

While we hypothesized that people have an underlying tendency to favor the lucky, we believe that adults override this tendency when they have the opportunity to do so, as they did in Experiment 1a. The lack of a tendency to favor the unlucky in Experiment 1b is consistent with our hypothesis that conscious, cognitively-demanding processes were at least partially responsible for the tendency to favor the unlucky over the lucky in Experiment 1a.

Importantly, the results on the intentional good and bad items in Experiment 1b demonstrate that participants were able to read and process the statements while under cognitive load, suggesting that the lack of a preference for lucky over unlucky (or unlucky over lucky) was not simply driven by participants’ failure to read and comprehend statements while performing the visual-spatial task. As further evidence that the load was not too challenging, on average, participants correctly identified whether the second stimulus was identical to the first 91% of the time (50% would be chance).

That said, because participants did not show a luck preference, but instead a lack of preference for the unlucky under load, their performance in this experiment is compatible with two different explanations. First, people may not be subject to the luck preference. Instead, their feelings of empathy or jealousy may lead them to favor the unlucky under conditions of low cognitive load, and these feelings may diminish or diminish in their impact as load increases. Second, adults may experience a luck preference as children do [6], but they both suppress it and override it under optimal cognitive conditions. In the present experiment, the cognitive load may have diminished the strength of this over-correction process, but it may not have been strong enough to eliminate that process altogether (the latter is supported by participants’ high performance on the memory task). Perhaps participants were able to engage in some controlled processes but not enough to completely override an underlying luck preference.

Experiments 2a and 2b distinguish these possibilities by taking a different approach to reduce the influence of conscious, deliberative processing on adults' evaluations of others, namely reducing explicit memory for the statements (c.f. [13], [16]). If adults prefer the unlucky because of automatically-induced empathy or jealousy, then they should continue to prefer the unlucky in Experiments 2a and 2b. In contrast, if explicit preference for the unlucky results from an explicit correction of an unconscious preference for the lucky, then when explicit memory is reduced or eliminated, a preference for the lucky should emerge. Conscious correction should not reverse this preference, because participants will fail to remember when it is necessary and when it is not.

## Experiments 2a and 2b: Effects of Delay on Evaluations of the Lucky and Unlucky

In Experiments 2a and 2b we tested evaluations of the lucky and unlucky after a delay to ask whether the luck preference exists in adults, but is masked in situations of uninhibited processing (Experiment 1a). Participants learned about good, bad, lucky and unlucky people without explicitly evaluating them. Then, after a delay during which we expected participants to forget which individuals were good, bad, lucky or unlucky, we tested whether participants would express a preference for lucky over unlucky (and good over bad) targets. Conceptually similar work has demonstrated that a valenced “tag” can be created upon hearing of intentional good and bad actors, and later retrieved in the absence of declarative memory for the events informing the evaluation [16]–[17].

There were a few reasons to investigate this question within a population of older adults (Experiment 2b) in addition to a younger adult sample (Experiment 2a) like the ones used in the previous two experiments. First, it seemed plausible that while a delay might impair younger adults’ memory, their memory might remain so strong that they would not reach chance performance. Older adults have worse episodic memory than young adults, and their memory for associative information is particularly poor (e.g., [28]). Thus, older adults were more likely to forget the particular actions or events associated with each person. Moreover, even when older adults retain those details, they can be less likely than young adults to focus on those details, and instead are more likely to rely on schematic, general information (e.g., [29]–[30]). Older adults are particularly likely to attend to the affective tone of information and to rely on affect as information when making decisions (e.g., [31]–[32]). Testing both younger adults and older adults allowed us to examine the hypothesis that adults show a luck preference, and to explore how an associative mechanism might explain such a preference.

### Methods

#### Participants

Fifty-four college student participants (*M* = 22 years old, *SD* = 5 years; 32 female, 22 male) completed Experiment 2a at a campus in the Northeastern United States in exchange for a small payment or for credit as part of a research requirement. An additional five participants were excluded: 2 did not complete all parts of the experiment, 2 were excessively fast in completing the tasks (leading us to believe they did not pay attention to the tasks), and one experienced a computer malfunction during the study. Experiment 2b included 24 older adults aged 53–84 (16 female; *M* = 70 years, *SD* = 8 years; *M* = 15 years of education, *SD = *2.7 years). The latter participants had previously completed a number of diagnostic health, mental health and cognitive tasks and none indicated signs of clinical depression (Beck Depression, *M* = 0.9, range 0–6), dementia (Mini-mental status examination, *M* = 29.3, range 27–30), or anxiety (Beck Anxiety, *M* = 3.9, range 0–9).

(For various reasons the sample size was much larger in Experiment 2a than in the other experiments in this paper. If, however, we examine only the first 24 participants (the same sample size as the Experiment 2b), the primary result–namely a significant preference for the lucky–still emerges *t*(23) = 2.95, *p* = .007).

#### Procedure

Experiment 2a began with an *encoding* phase in which participants saw 40 photographs, each paired with a unique statement (from Experiment 1a) for 10 seconds, and were asked to form an impression of each person. This phase was nearly identical to that in Experiment 1a; the only difference was that participants were not asked to make an evaluation after forming each impression. (Note, one lucky event in Experiment 2a was significantly less preferred than all other lucky events, all *p*s<.002, and was therefore excluded from all analyses. This item was replaced in Experiments 1a, 1b and 2b (which were all run *after* Experiment 2a) with a different, equally rated, lucky item. No other item was significantly more liked or disliked than all other items in the same category.).

After encoding, participants completed 20 minutes of filler tasks (e.g., sudoku puzzles). Next, participants completed a *face recognition task* in which they indicated whether each of 80 faces (40 old, 40 new) had been seen in the encoding phase or not. The 40 photographs that were old for half of participants were new for the other half of participants and vice versa for the other 40 photographs. Then participants completed an *evaluative rating task* in which they saw the 40 faces viewed during the encoding phase along with 10 novel faces (included so that we could tell them that if the face looked unfamiliar they could just guess at an evaluation) and rated their preference for each face on a 6-point scale (with higher numbers signifying greater liking). Finally, participants completed a *memory test* in which they viewed the faces from the encoding phase and chose which of the four categories of statements had been originally paired with the face (good action/bad action/lucky event/unlucky event; note that this was the first time these four categories were explicitly introduced). The order of presentation of faces varied for each task, and two versions of each task were used to protect against potential effects of order.

The procedure in Experiment 2b (older adults) was identical to the procedure from Experiment 2a (younger adults) with two exceptions. For the older adults, five neutral practice face+statement pairs were included prior to encoding to be certain that each participant had sufficient time to read the statement before the slides forwarded automatically. In the rare case that a participant was unable to read these items quickly enough (N = 4), the participant was administered a self-paced version of the encoding task. Also, no new faces were presented in the evaluative rating task in order to shorten the task for older participants.

### Results: Experiment 2a

#### Explicit memory

Participants recognized the faces of individuals from the encoding task as familiar more often than expected by chance; that is, they correctly recalled which faces were new more often than they mistakenly labeled new faces as old, *M* = 0.49, *t*(53) = 15.61, *p*<.001 for lucky/unlucky items and *M* = .53, *t*(53) = 17.56, *p*<.001 for good/bad items, one-sample t-test compared to 0. Participants had fairly poor memory for the type of event (e.g., lucky) associated with a particular target, though rates of recognition were significantly above chance: for intentional good/bad items (*M* = 36% correct, chance = 25%), *t*(53) = 7.25, *p*<.001, and for lucky/unlucky items (*M* = 29% correct, chance = 25%), *t*(53) = 2.72, *p* = .009.

#### Omnibus ANOVA

We next computed a 2 (valence: positive vs. negative) X 2 (intentionality/controllability: intentional/controllable vs. unintentional/uncontrollable) repeated-measures ANOVA as we had in Studies 1a and 1b. In Study 2a we found a significant effect of valence such that positively-valenced items were rated more positively than negatively valenced items, *F*(1, 53) = 10.26, *p* = .002. In contrast to the previous studies we found no significant difference in the treatment of intentional/controllable vs. unintentional/uncontrollable items, *p* = .130, and no significant interaction between valence and intentionality/controllability, *p* = .663.

#### The luck preference

Analyses parallel to those from Experiments 1a and 1b were conducted on the liking ratings to assess whether participants showed a preference for the (un)lucky. In contrast to Experiments 1a and 1b, participants, after a delay, preferred the lucky (*M* = 3.57) to the unlucky (*M* = 3.42), *t*(53) = 2.26, *p* = .028, see Table 1. They also preferred intentional good actors (*M* = 3.52) to intentional bad actors (*M* = 3.34), *t*(53) = 2.39, *p* = .02.

#### Relationship between liking and memory

Importantly, there was no relationship between the extent of the luck preference (M_lucky_ – M_unlucky_) and performance on the type of memory task, *r* = .20, *p* = .15, demonstrating that there was no relationship between an individual’s memory performance for who experienced which type of event and their overall tendency to favor the lucky over the unlucky. Interestingly, those with particularly bad explicit memory did not show more of a luck preference, perhaps because no participants showed particularly strong memory and therefore no one could reliably use memory in forming evaluations. Instead, participants likely formed affective associations with the faces, and used the stored affective information in their explicit evaluations of the faces 20 minutes later.

### Results: Experiment 2b

#### Explicit memory

The older adult participants were also able to recognize individuals as familiar or not, as indicated by their relatively high performance on the face recognition task (hits minus false alarms, *M* = 0.35), *t*(23) = 6.62, *p*<.001, one sample t-test. However, these participants had considerable trouble recalling the type of event associated with each target, as indicated by performance not significantly different than chance on the memory task (*M* = 28.0%, chance = 25%), *t*(23) = 1.70, *p* = .10, one-sample t-test. Participants were not significantly better at recalling the type of event associated with the intentional good/bad items (*M* = 29% correct) than the lucky/unlucky items (*M* = 27% correct), *p*>.25, though performance was marginally better than chance for intentional good/bad items, one sample t-test, *t*(23) = 1.96, *p* = .06, and was not significantly different from chance for lucky/unlucky items, *p*>.40.

#### Omnibus ANOVA

In Study 1b, participants showed a main effect for valence such that they preferred positively-valenced items to negatively-valenced ones, *F*(1, 23) = 32.34, *p*<.001. Participants showed no significant effect of intentionality/controllability, *F*(1,23) = .91, *p* = .349. Finally, there was a significant interaction between valence and intentionality/controllability, *F*(1,23) = 4.45, *p* = .046, suggesting that the difference between positively- and negatively-valenced items was smaller in the case of unintentional/uncontrollable items than intentional/controllable ones.

#### The luck preference

Like their younger counterparts, older adults also preferred the lucky (*M* = 3.95) to the unlucky (2.74), *t*(23) = 5.18, *p*<.001, see Table 1, and preferred intentional good actors (4.05) to intentional bad actors (2.47), *t*(23) = 5.53, *p*<.001. In fact, older adults’ preferences for the lucky over the unlucky and their preference for intentional good over bad actors were larger than younger adults’ preferences, equal variances not assumed, *t*(26.40) = 4.42, *p*<.001, and, *t*(26.40) = 4.72, *p*<.001, respectively. Older adults showed a slightly larger good preference than luck preference, *t*(23) = 2.11, *p* = .046.

#### Relationship between liking and memory

As was the case with younger adults, memory for the type of event associated with each face was not significantly related to the luck preference index, *r* = .15, *p*>.45, providing additional evidence of the dissociation between remembering specifics about the targets and evaluations of the targets.

### Discussion

Both younger and older adult participants, after a delay, preferred lucky to unlucky individuals, despite the fact that they had poor memory for who was actually lucky and unlucky. This finding is the *opposite* of the finding in Experiment 1a, in which younger adults explicitly preferred the unlucky to the lucky when they evaluated individuals with no delay (or load). This shift in evaluations provides initial evidence that adults prefer the lucky to the unlucky–but under conditions of unlimited time and cognitive resources, they report a preference for the unlucky because of other consciously-controlled processes.

That said, the actual effect sizes in the memory studies, especially in the younger adult sample, were fairly small. This occurred, we suspect, because adults were given the somewhat awkward task of coming up with an explicit evaluation of people about whom they could recall almost nothing. It is more surprising that any differences did emerge, and it would be unreasonable to expect these evaluations to be as large in absolute magnitude as some of the effects in the immediate evaluation condition.

Interestingly, while both older and younger adults relied heavily on valence in their evaluations, this difference was noticeably greater in older adults. This greater reliance on valence is likely to be explained by some combination of older adults’ greater reliance on schematic, general information rather than details [29]–[30] and their increased reliance on affective information when making decisions [31]–[32]. In other words, by creating a general gist and using affect more as the basis of on-line judgments, the older adults presumably had a stronger affective tag to rely on when they were asked to make evaluations 20 minutes after the initial impressions were formed. Preference for the lucky (and for the good) may be particularly likely to be revealed in situations where people are relying on the affective tag associated with an item, and when they have little memory of how that tag was created. Older adults may achieve both of these criteria more readily than younger adults.

## General Discussion

Across two experiments we demonstrated an intriguing dissociation between evaluations of the lucky and evaluations of the unlucky. Participants reported that they preferred the unlucky when asked to evaluate lucky and unlucky targets immediately after encountering them, but showed no preference under cognitive load, and actually preferred the lucky when evaluations were obtained after loss of explicit memory. That is, when participants were asked to evaluate lucky and unlucky targets in a simple questionnaire they expressed a greater liking of unlucky victims over lucky beneficiaries. However, when their ability to engage in controlled processes was limited, this espoused preference did not appear. Finally, when participants learned about lucky and unlucky individuals, but refrained from explicit evaluation long enough to allow explicit memory loss, they later preferred the lucky to the unlucky. Similar to the performance of amnesic patients in previous affective judgment tasks for good versus bad actions [16]–[18], healthy adults’ lingering preference for lucky over unlucky targets reveals their ability to retain knowledge of the affective valence of the associated information, despite losing much of the explicit memory for the event itself.

One potential explanation for participants’ preference for the lucky is Just World Theory [2] which explains that in order to deal with the threatening, chaotic, and unpredictable world, people manage their anxiety with beliefs such as “good things happen to good people” and “bad things happen to bad people.” Because people see themselves as good, they don’t need to live in fear of bad events occurring, and the world feels less threatening. The insidious effect of this belief is that when an otherwise innocent person is observed to experience an unfair or otherwise negative event, the viewer changes his/her evaluation of the person from innocent to bad, thereby protecting the viewer’s sense that the world is just. If these beliefs are activated in initial impression formation, leading to evaluations that are retained over time, then Just World Theory could help explain why adults favored the lucky over the unlucky in the memory experiments.

Another explanation for adults’ preference for the lucky, very likely in concert with just world beliefs, is an associative one. A century of work has demonstrated the power of associative learning, or the acquisition of binding between two mental concepts [33]–[34]. The constraints and complexities of associative learning have been studied in humans and non-human animals (for reviews see [35]–[37]) and such learning is believed to be a central mechanism of learning and memory. More recently, a specific type of associative learning called *evaluative conditioning* has demonstrated that neutral stimuli can come to be valenced simply by their association with a valenced object, face, taste or scent, after as little as one instance of association (for a review see [38]). The luck preference could be explained by this type of “affective tagging.” That is, the positivity or negativity of the lucky or unlucky event could rub off on the target, resulting in a more positive evaluation of lucky individuals and a more negative evaluation of unlucky individuals.

There are many other cases of “affective tagging” or cases in which affective information associated with a person, event or object “rubs off” on evaluations of another person, event or object, in the literature (e.g., [39]–[40]). For example, adults tend to dislike an individual if she bears information they disagree with, and this dislike emerges even when the bearer herself disagrees with the information she is sharing [41]. This generalization suggests that the evaluation of the information has rubbed off on the bearer of the information. Similarly, people dislike a sweater previously owned by someone with AIDS or previously worn by someone in a car accident (that was not his fault), compared to one owned by a healthy man with no accident history, even when they are told that the sweater has been thoroughly cleaned [42], suggesting that affect may have traveled from the owner to the object. Affect may not only spread from an event, a physical object, or information to a person, but from one person to another. For example, adults see an individual as more angry if he/she has described another person as angry [43]. Similarly, children and adults have shown a tendency to dislike an average-weight individual more if he is merely next to an obese person in a waiting room compared to when he is next to an average weight person ([44]; for related work on *associative stigma* see [45]). These demonstrations suggest that a spatially *or* psychologically contemporaneous location can lead to affective spreading across individuals, providing evidence of affective tagging in other domains.

Evidence consistent with affective tagging was observed in Experiments 2a and 2b, in which participants continued to prefer lucky to unlucky individuals even though they had largely forgotten whether the specific individuals had performed intentional good or intentional bad actions or had experienced lucky or unlucky events. A simple and parsimonious mechanism like affective tagging can also explain why the luck preference appears by 3 years of age and in diverse cultures [3]. Even at a young age, children know that some events are good and others are bad, and once these evaluations can be made, the target of the event may be similarly branded as good or bad.

These studies also contribute to an emerging set of results that suggest that adults may continue to hold many of the same biases or attitudes that young children do, but unlike children, they appear to have the motivation and capacity to correct these. For example, Epley, Morewedge & Keysar [46] found that adults show the same egocentric biases in perspective-taking as children, but that they correct these biases in ways that children do not. More generally, these results support the view that remnants of early social and non-social cognition likely lie just under the surface of adult social and non-social cognition [46]–[48].

Our results suggest that at the moment of meeting lucky and unlucky individuals, we may be able to consider that they did not play a role in causing these events, and therefore discount the information associated with them (including valence), but when we fail to engage in this discounting due to distraction or delay, we rely on the valence of the event more. While we may clearly distinguish between a lottery winner and a saint in the moment, months later, we may not.

## Supporting Information

Appendix S1 Items used in all experiments.
(DOCX)
