# Supplementary material for: The Role of Forgetting in Undermining Good Intentions
Source: PLoS One. 2013 Nov 13;8(11):e79091. doi: 10.1371/journal.pone.0079091 (PMC3827336; doi:10.1371/journal.pone.0079091)
Supplement: Appendix S1 — Items used in all experiments. (DOCX) [file pone.0079091.s001.docx]

Appendix S1

**Bad**

1. Last year Dennis burned down a neighbor’s shed because he was mad at him.
2. Last weekend, Russell saw an unlocked bike outside a library and took it home to keep and ride.
3. Last week Gabriel cheated on a college entrance exam using his cell phone.
4. Chad made up a list of important-sounding jobs to list as experience on a resumé in order to get a high-paying position.
5. Matt left the restaurant before paying the bill.
6. Gerald parked in a handicap spot and used his grandfather’s placard even though he wasn’t there.
7. Andy avoided helping his partner find the right answer so that he would be the only one to get it right.
8. Arthur crawled under the toll gate to avoid paying.
9. Evan fed his infant son brandy to quiet him on the plane.
10. Today as Darrin left the living room, he took the remote so that his brother couldn’t easily watch his show.

**Good**

1. Dwight donated one of his kidneys to his neighbor.
2. Daniel volunteered for the Peace Corps.
3. Greg bought lunch for his officemate because she was having a bad day.
4. Knowing his neighbors had a newborn, Sam shoveled the snow off their sidewalk while shoveling his own.
5. Leonard pulled over to help a motorist with his stalled car.
6. Mike donated his old books to the local high school.
7. Keith painted a picture for his bedroom wall.
8. Bob climbed the tree to retrieve the kitten.
9. Trevor helped his neighbor jump-start her car.
10. Brian took out the recycling and biked to work.

**Lucky**

1. Will won a drawing for a brand-new car.
2. Last week Tom was at the grocery store when they announced that he was the millionth customer and had won free groceries for a year.
3. Tony inherited $100,000 from a great-aunt he’d never met.
4. When Sean went to pay for his book, it turned out to be 75% off of the marked price.
5. On a recent plane ride Chris was assigned to sit next to his celebrity crush who spoke with him for the 6 hour plane ride.
6. A new restaurant opening in Jack’s neighborhood sent him a gift certificate for a free meal as a promotion.
7. Lloyd’s favorite cake was on sale at the bakery this week.
8. Stephen put money in a vending machine and two candy bars came out instead of one.
9. Eugene’s arms were full of groceries but someone walked out just as he got to the door, so he didn’t have to take out his keys. (original in Study 2a) Eugene found his favorite aftershave for 50% off at the drugstore when he went there this morning. (replacement for Studies 1a, 1b, and 2b)
10. Andrew was walking when he found $5 on the ground.

**Unlucky**

1. Derek was excited to go home for the holidays, but both his flights were cancelled and so he spent Christmas in the airport.
2. At Albert’s recent annual physical exam, he was diagnosed with a rare form of pancreatic cancer.
3. Nelson walked outside to discover that a shopping cart had smashed into the side of his new car.
4. A month ago Todd’s house was destroyed in a tornado.
5. Last week Glen’s childhood dog, whom he’d taken care of all his life, died unexpectedly of natural causes.
6. Brett’s birthday gift was lost in the mail.
7. Richard was stuck in the elevator for three hours with ten other people.
8. Anthony left the store to find that someone had double-parked in front of his car.
9. The snack machine ate Christian’s dollar without giving him credit.
10. Martin woke up to see his car was covered in 3 feet of snow.
